# Characterization of two antimicrobial peptides produced by a halotolerant *Bacillus subtilis* strain SK.DU.4 isolated from a rhizosphere soil sample

**DOI:** 10.1186/2191-0855-3-2

**Published:** 2013-01-05

**Authors:** Piyush Baindara, Santi M Mandal, Niharika Chawla, Pradip Kumar Singh, Anil Kumar Pinnaka, Suresh Korpole

**Affiliations:** 1 MTCC and Gene Bank, CSIR-Institute of Microbial Technology, Sector 39A, Chandigarh, 160036, India; 2 Central Research Facility, Indian Institute of Technology Kharagpur, Kharagpur, West Bengal, 721302, India

**Keywords:** *Bacillus*, Antimicrobial peptide, Lipopeptide, Chromatography, RP-HPLC, MALDI

## Abstract

A bacterial strain producing two antimicrobial peptides was isolated from a rhizosphere soil sample and identified as *Bacillus subtilis* based on both phenotypic and 16S rRNA gene sequence phylogenetic analysis. It grew optimally up to 14% NaCl and produced antimicrobial peptide within 24 h of growth. The peptides were purified using a combination of chemical extraction and chromatographic techniques. The MALDI-TOF analysis of HPLC purified fractions revealed that the strain SK.DU.4 secreted a bacteriocin-like peptide with molecular mass of 5323.9 Da and a surface-active lipopeptide (m/z 1056 Da). The peptide mass fingerprinting of low-molecular-weight bacteriocin exhibited significant similarity with stretches of secreted lipoprotein of *Methylomicrobium album* BG8 and displayed 70% sequence coverage. MALDI MS/MS analysis elucidated the lipopeptide as a cyclic lipopeptide with a β-hydroxy fatty acid linked to Ser of a peptide with seven α-amino acids (Asp-Tyr-Asn-Gln-Pro-Asn-Ser) and assigned it to iturin-like group of antimicrobial biosurfactants. However, it differed in amino acid composition with other members of the iturin family. Both peptides were active against Gram-positive bacteria, suggesting that they had an additive effect.

## Introduction

Antimicrobial peptides characterization has received great attention in the recent past due to their applications as food preservatives without any toxic effects on host and therapeutic agents. At present preservation of food is a serious concern for almost all countries across the world. Since lactic acid bacteria (LAB) produce an array of antimicrobial substances they were used as natural bio-preservatives for special applications (Holzapfel et al. 1995; Cotter et al. 2005; Deegan et al. 2006). Other bacteria such as *Enterococcus*, *Streptococcus* etc., were also reported to produce various bacteriocins and they are also being considered for different applications. However, production of antimicrobial peptides by *Bacillus* strains has been increasingly characterized in the recent past and many peptides produced by this group of bacteria found to be suitable for various applications (Abriouel et al. 2011). The antimicrobial peptides produced by *Bacillus* spp., includes various classes of bacteriocins (Klaenhammer 1993), antimicrobial surface-active biosurfactants like lipopeptides, glycopeptides and nonribosomally synthesized cyclic peptides (Mukherjee et al. 2006; Rodrigues et al. 2006). Among the antimicrobial biosurfactants, lipopeptides contain peptides with 7–10 amino acid that are cyclised via a lactone ring to a β-hydroxy fatty acid with different chain lengths. The lipopeptides produced by various *Bacillus* sp., are further divided into different classes such as iturins (Delcambe et al. 1977), surfactins (Arima et al. 1968), fengycins (Vanittanakom et al. 1986), kurstakins (Hathout et al. 2000), bacillomycins (Roongsawang et al. 2002) and mycosubtilin (Duitman et al. 1999). Among these, iturins are the most widely reported class of lipopeptides produced by multiple strains of *Bacillus subtilis* (Duitman et al. 1999; Isogai et al. 1982; Peypoux et al. 1986), *B*. *licheniformis* (Kakinuma et al. 1969; Yakimov et al. 1999; Jenny et al. 1991; Lin et al. 1994) and *B*. *cereus* (Nishikiori et al. 1986). The lipopeptides like surfactin or iturin are synthesized by multifunctional enzymes encoded by large gene clusters (Kleinkauf and von Döhren 1995; Marahiel et al. 1997) and exhibit large diversity. Despite the fact that multiple bacteriocins and/or lipopeptide analogues are produced by a single strain, only few antimicrobial peptides are reported in the literature. Considering the enormous diversity of *Bacillus* species in the soil ecosystem it is very important to isolate and preserve the strains producing these antimicrobial peptides followed by detailed characterization. As a part of screening for biotechnologically potential soil microflora, we have earlier described a novel bacteriocin produced by a *Brevibacillus laterosporus* strain GI-9 (Singh et al. 2012). In present study we report another antimicrobial peptide and a cyclic lipopeptide produced by a halotolerant isolate of *Bacillus subtilis* strain SK.DU.4 isolated from a rhizosphere soil sample.

## Material and methods

### Isolation of bacteria and identification

The bacterial strain SK.DU.4 was isolated from a farmland soil sample. The sample was serially diluted and plated on different media to screen the bacteria producing antimicrobial substances. Selected colonies were streaked on to nutrient agar (NA) medium with the following composition (g/l): peptic digest of animal tissue, 5.0; beef extract, 1.5; yeast extract, 1.5; sodium chloride, 5.0; agar 15.0 and the pH adjusted to 7.2. The isolates were checked for purity and preserved at −70°C for further studies. The indicator strains used in this study were obtained from Microbial Type Culture Collection and Genebank (MTCC), Chandigarh, India and grown on tryptone soya agar (TSA) medium with the following composition (g/l): pancreatic digest of casein, 15.0; papaic digest of soybean meal, 5.0; sodium chloride, 5.0; agar 15.0 and the pH adjusted to 7.2. Strain SK.DU.4 was tested for various phenotypic properties including morphology, physiology and biochemical characteristics, according to the standard procedures. To confirm the identification of strain SK.DU.4, the 16S rRNA gene was amplified by PCR using universal primers and the amplified PCR product was sequenced as described earlier (Suresh et al. 2006). The 16S rRNA gene sequences of closely related strains were retrieved from EzTaxon server and aligned using CLUSTAL_W program of MEGA version 5.0 (Tamura et al. 2011). The alignment was corrected manually using BioEdit sequence alignment editor (Hall, 1999). Upon calculating the pair-wise evolutionary distances (Kimura, 1980), a neighbour-joining phylogenetic tree was constructed using the MEGA version 5.0.The strain was deposited at Microbial Type Culture Collection and Genebank, (MTCC 11460) and the 16S rRNA gene sequence was submitted to EMBL (HF544505) database.

### Determination of bacteriocin activity

The bacteriocin activity was determined by well diffusion assay. The strain was grown for 24 h in 200 ml nutrient broth (NB, Himedia) using 500 ml flask and subsequently cells were removed by centrifugation (8000 rpm for 20 min, 4°C). The supernatant obtained was filtered by using 0.22 μm filter (Millipore), diluted by two fold and used to test the activity as mentioned earlier (Singh et al. 2012). The antimicrobial peptide production was also tested by growing the strain in minimal medium. To examine the antimicrobial activity, *S*. *aureus* cells were processed for scanning electron micrograph (SEM) as described by earlier (Singh et al. 2012). Growth curve was prepared to examine the bacteriocin production along with the growth of strain SK.DU4.

### Production and purification of bacteriocin

To test the effect of different carbon and nitrogen sources like glucose, lactose, yeast extract, peptone and beef extract on production of antimicrobial peptides, 0.5% of each of them added to minimal medium with following composition (g/l): Na_2_HPO_4_.2H_2_O, 7.9; KH_2_PO_4_, 3.0; NaCl, 0.5; NH_4_Cl, 1.0; pH 7.2. The antimicrobial peptides production was measured as zone of inhibition against indicator strains. For the characterization of peptides, culture was grown in 1000 ml of NB using 2 l flasks to obtain large quantity of antimicrobial peptides. One percent (v/v) of overnight grown culture of SK.DU.4 was inoculated to 1 l of NB medium and grown for 24 h on a rotary shaker at 30°C. Subsequently cells were separated by centrifugation (8000 rpm, 20 min at 4°C). The supernatant was mixed with 2% (w/v) of Diaion HP-20 (Supelco, SigmaAldrich, USA) resin and the antimicrobial peptide was eluted in methanol. Methanol was evaporated using a rota vapour (BUCHI Rota vapor R-200), the antimicrobial peptide was redissolved in Milli-Q water and further purification was achieved through reverse phase HPLC (1260 Infinity, Agilent Technologies, USA) using a semi-preparative C18 column (Pursuit 10C18 250×21.2 mm). Acetonitrile and 0.1% TFA were used as mobile phase. The purified bacteriocin was tested for antimicrobial activity and applied on Tricine-SDS-PAGE (16%)/6 M urea.

### Mass spectrometry and peptide mass fingerprint analysis

Matrix-assisted laser desorption ionization (MALDI) was used to primarily characterize the antimicrobial bacteriocin. The lyophilized peptide was re-suspended in methanol and 4 μl of peptide solution was mixed with 4 μl of matrix (CHCA, 10 mg/ml), 1.0 μl of this mixture solution was spotted onto the MALDI 100 well stainless steel sample plate and allowed to air dry prior to the MALDI analysis (Mandal et al. 2009). To obtain MALDI mass spectra, a Voyager time-of-flight mass spectrometer (Applied Biosystem, USA), equipped with 337 nm N_2_ laser was used and operated in accelerating voltage 20 kV. The spectra were recorded in positive ion linear mode. Reproducibility of the spectrum was checked several times from separately spotted samples. In order to carry out peptide mass fingerprint (pmf) analysis of the purified bacteriocin solution, the sample was lyophilized and dissolved in 50 mM ammonium bicarbonate (pH 8.0) buffer. Protein was reduced with 0.1 M DTT at 56°C for 30 min and alkylated with 0.3 M iodoacetamide at room temperature for 15 min in the dark as described by Dey et al. (2012). Subsequently, the sample was subjected to digestion with trypsin at 37°C for overnight. One microliter of the digested sample was spotted directly onto a MALDI target plate and 1 μl of CHCA matrix solution was applied on the sample spot and allowed to air dry. Mass spectra of different peptide fragments were acquired in positive ion reflector mode with a mass range of 700–5000 Da after an average of 1000 laser shots. Data was uploaded into MASCOT (Matrix Science, London, UK) database to search the protein identity. During database search the following parameters were considered: database, NCBInr; maximum missed cleavages, 1; precursor tolerance, 0.2 Da; peptide charges, +1; mass, monoisotopic.

### In-gel activity assay for detection of bacteriocin activity

Tricine-sodium dodecyl sulfate–polyacrylamide gel electrophoresis (Tricine-SDS-PAGE) was performed for antimicrobial peptide using 16% polyacrylamide gel. The purified and concentrated antimicrobial peptide was applied onto the gel in duplicate. After the electrophoresis, a part of the gel along with molecular weight markers was stained with Coomassie Brilliant Blue R-250 to visualize the protein bands. Whereas the unstained part of the gel was used for in situ detection of bacteriocin activity upon fixing it in a mixture of 2-propanol, acetic acid and H_2_O (25:10:65) for 15 min and washed with sterile H_2_O for 30 min repeatedly. It was placed in a sterile Petri dish, overlaid with 10 ml of soft agar (0.8%) containing test strain *S*. *aureus* (about 10^6^ cells/ml).

### Determination of minimum inhibitory concentration

The MIC of antimicrobial peptide was evaluated for different strains by using a microtiter plate dilution assay. Test strains grown to logarithmic phase under optimal conditions (between 0.3-0.4 OD) and used for assay in triplicates as described earlier (Singh et al. 2012). The lowest concentration that inhibited growth of test strain and did not show any increase in absorption after 48 h incubation was considered as MIC.

### Effect of pH, temperature and hydrolytic enzymes on bacteriocin activity

The antimicrobial peptide was checked for tolerance to different temperature, pH and proteases activity. To determine the tolerance to temperature and pH, the purified peptide was incubated at different temperatures such as 60°, 80°, 100° and 121°C for 15 min and pH values between 2.0–12.0. Different proteolytic enzymes including pepsin, trypsin, chymotrypsin, proteinase K and pronase E were incubated with peptide for 6 h at 37°C and the activity was terminated by heating at 80°C before the peptide was used for assay.

### Extraction of lipopeptide

Lipopeptide was isolated from bacterial culture in a combination of acid and solvent extraction procedure following Vater et al. (2002). In brief, cells were removed from the 24 h growing culture broth by centrifugation (13,000 × g) for 15 min at 4°C. The supernatant was adjusted to pH 2.0 by addition of concentrated HCl and allowed to precipitate at 4°C for 16 h. Precipitate was collected after centrifugation (13,000 × g) for 20 min at 4°C and extracted with methanol by stirring for 2 h. The lipopeptide containing methanol was collected after filtration and vacuum-dried.

### Purification of lipopeptide

The extracted lipopeptide dissolved in methanol and fractionated by reverse phase- HPLC (Agilent 1100 series) with a ZORBAX 300-SB18 column (4.6 mm × 250 mm, particle size 5 μm), at a flow rate of 1 ml/min. The solvent system used was 0.1% aqueous TFA (A) and acetonitrile containing 0.1% TFA (B). The gradient of solvent B used to run the column was as follows: 0-60% for 0–45 min, 60-80% for 45–55 min and 80-100% for 55–60 min. The elution from the column was monitored at 215 nm in a diode array detector and all the peaks of HPLC chromatogram were collected using a fraction collector (GILSON, France) coupled with the system. Collected fractions were concentrated by speed vacuum and antimicrobial activity was screened. The major fraction showing antibacterial activity was re-chromatographed in the same column under similar conditions, but solvent B was used as 100% ACN with a gradient of 0-10% for 30 min. Peptide concentration was determined using the RP-HPLC conditions and calibrated with surfactin (Sigma-Aldrich).

### MALDI-TOF-MS and sequencing

The molecular mass of the purified active lipopeptide fraction was analysed following the protocol described above. For MS/MS sequencing, the peptide was incubated with 10% NaOH in methanol at room temperature for 16 h to cleave the lactone ring. The cleaved peptide was lyophilized, again extracted with methanol and allowed for mass spectrometry analysis. The spectra were recorded in the post-source decay (PSD) ion mode as an average of 100 laser shots with a grid voltage of 75%. The reflector voltage was reduced in 25% steps and guide wire was reduced 0.02–0.01% with an extraction delay time of 100 ns.

### Fatty acid analysis by GC-MS

Acid hydrolysis of lipopeptide was performed by incubating the peptide (5 mg) with 0.5 ml of 6 M HCl at 90°C for 18 h in sealed tubes. The fatty acids were extracted with ether and esterified with 0.95 ml methanol and 0.05 ml of 98% H_2_SO_4_ at 65°C for 6 h. Fatty acid methyl esters were obtained after extraction with n-hexane and analyzed by GC-MS with a Clarus 500 GC (PerkinElmer, USA). Helium was used as carrier gas at a flow rate of 1.0 ml/min. The column temperature was maintained at 120°C for 3 min and thereafter gradually increased (8°C/min) to 260°C.

## Results

### Characterization of bacterial strain

**Figure 1:**
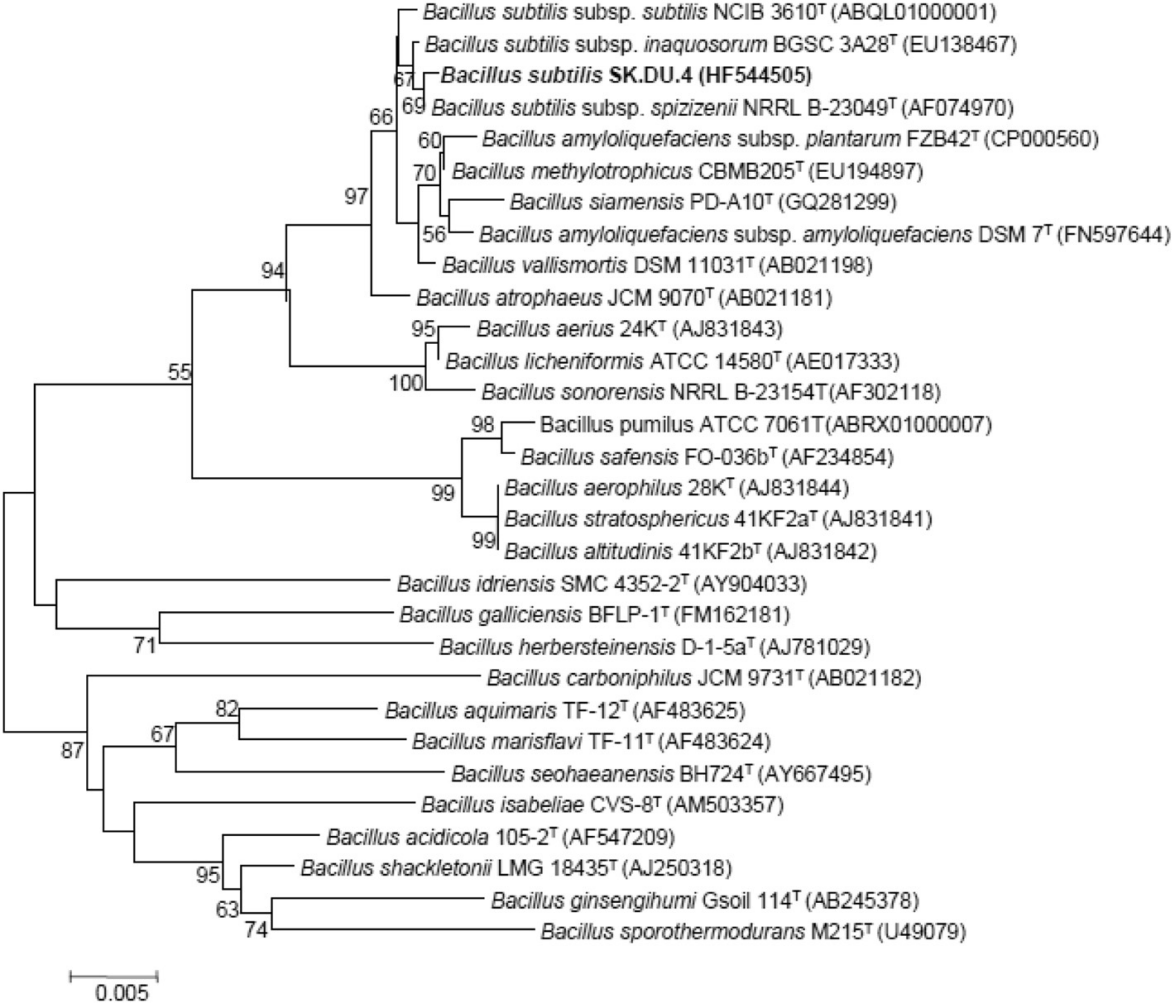
**Neighbour-joining phylogenetic tree based on 16S rRNA gene sequences, showing the phylogenetic relationship between strain SK.DU.4 and other members of the genus *Bacillus*.** Bootstrap values greater than 50% are given at the nodes.

**Figure 2:**
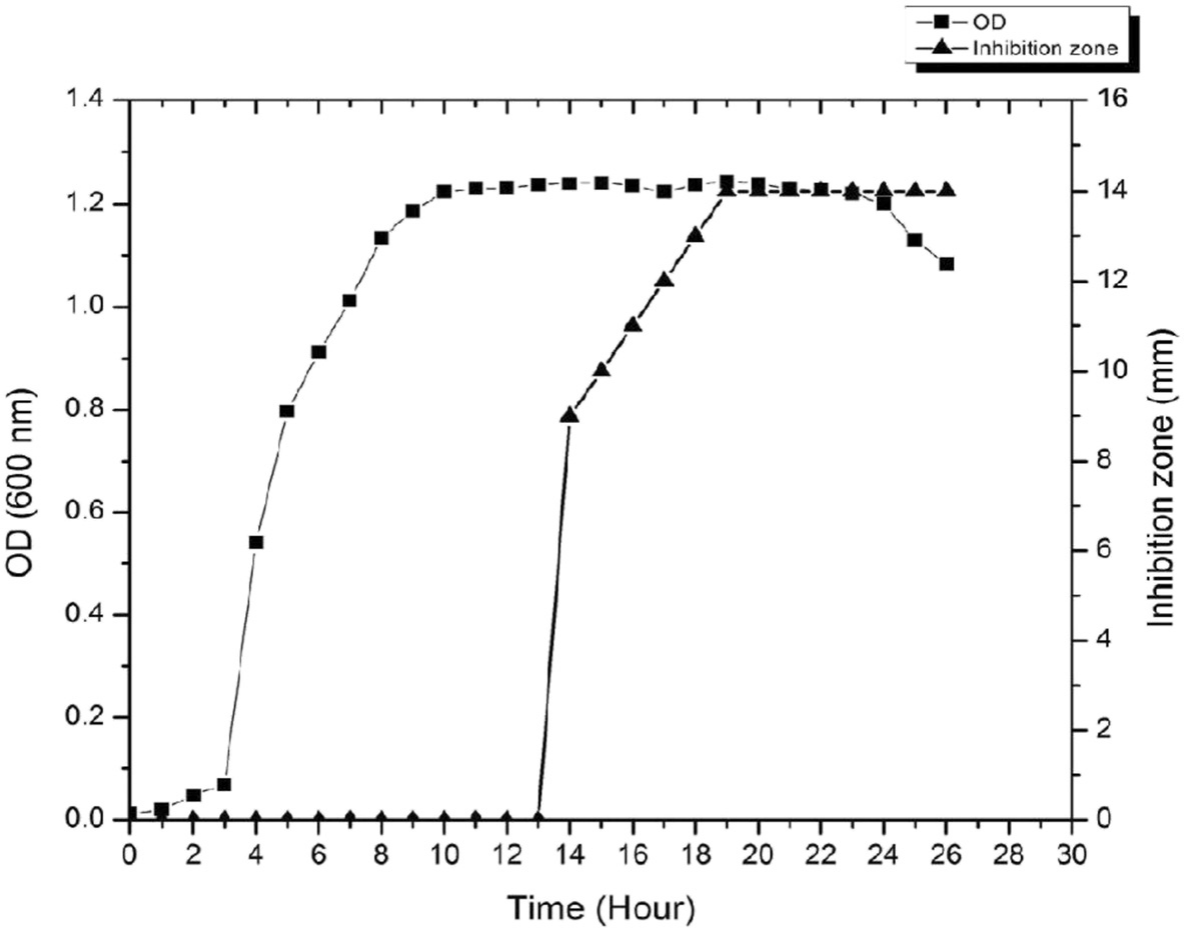
**Growth phase-dependent bacteriocin production by *Bacillus subtilis *strain SK.DU.4.** While *filled squares* represent bacterial growth as measured by absorbance at 600 nm, *filled triangles* indicate corresponding bacteriocin activity as determined by inhibition zone assay.

A strain, designated SK.DU.4, has been shown to exhibit antibacterial activity as revealed by the clearing zone assay while screening. To this effect, we sought to investigate the molecular basis of its antimicrobial activity. Initial efforts to identify the strain by phenotypic characteristics revealed that the strain SK.DU.4 was a Gram-positive, rod shaped, endo-spore forming bacteria with positive reaction for catalase activity and negative for oxidase activity. It produces amylase and proteases as revealed by starch and skim milk hydrolyses. It grew between 10° to 50°C temperature. Positive for methyl red and nitrate reduction but negative for indole test and urea hydrolysis. All these phenotypic properties are in agreement with members of the genus *Bacillus*, known to produce diverse antimicrobial peptides. In addition to the phenotypic identification, the BLAST analysis of 16S rRNA gene sequence revealed significant identity (99.8%) with *B*. *subtilis* subsp. *inaquosorum*, a strain shown to produce surfactin-like lipopeptide. Further, the neighbour joining phylogenetic tree constructed with 16S rRNA gene sequences of other members of the genus *Bacillus* confirmed that strain SK.DU.4 belongs to genus *Bacillus* as it formed a distinct cluster along with other subspecies of *B*. *subtilis* (Figure 1). Although strain SK.DU.4 showed highest similarity with *B*. *subtilis* subsp. *inaquosorum*, it formed a clade with *B*. *subtilis* subsp. spizizenii, with low bootstrap value. Therefore, strain SK.DU.4 was assigned as another strain of *B*. *subtilis*. The growth curve analysis along with studies on antimicrobial production indicates that the strain SK.DU.4 also produces a peptide like antimicrobial substance during stationary growth phase (Figure 2). Notably, there is a significant increase in antimicrobial peptide production during 14 to 18 h and the antimicrobial activity remained constant thereafter as measured by inhibition zone.

### Production and purification of antimicrobial peptide

Although the antimicrobial peptides were produced in NB for large scale preparation, the influence of different carbon and nitrogen sources on peptides production was tested using minimal medium. As revealed by inhibition zone, increased production of antimicrobial peptides was observed with addition of beef extract (15 mm) followed by the combination of glucose and yeast extract (14 mm), and peptone (13 mm) after 24 h. The optimum pH for the production of antimicrobial peptides was 7.2 and there was no significant change in pH was observed during the production of antimicrobial peptides. The bacteriocin-like peptide was extracted from CFB by hydrophobic interaction chromatography using Diaion HP-20 beads and purified by a combination of chromatography techniques as described previously (Singh et al. 2012). Subsequently, the purified peptide was tested for antimicrobial activity against an array of Gram-positive and Gram-negative bacteria, yeasts and fungi strains using well diffusion assays. Interestingly, the antimicrobial peptide shows antibacterial activity against Gram-positive bacteria only, without a detectable effect against yeast (Table 1). The influence of medium composition for antimicrobial activity of the peptide was ruled out as comparable results of antimicrobial activity of strain SK.DU.4 obtained while grown on minimal medium.

**Table 1:** Inhibition spectrum of bacteriocin-like peptide and lipopeptide produced by strain SK.DU.4

**Indicator species**	**Strain**	**Inhibition activity**		
		**Bacteriocin-like peptide**	**Lipopeptide**	**Polymyxin B (300 U)**
*S*. *aureus*	MTCC1430	**+**	**+**	**+**
*B*. *subtilis*	MTCC121	**-**	**+**	**+**
*B*. *subtilis*	MTCC441	**-**	**-**	**+**
*B*. *subtilis*	MTCC619	**-**	**-**	**+**
*B*. *subtilis*	MTCC736	**-**	**+**	**+**
*B*. *subtilis*	MTCC1427	**-**	**+**	**+**
*B*. *subtilis*	MTCC2423	**-**	**+**	**+**
*B*. *subtilis*	MTCC9878	**-**	**+**	**+**
*L. monocytogenes*	MTCC839	**+**	**+**	**+**
*S*. *mutans*	MTCC497	**+**	**-**	**+**
*M*. *luteus*	MTCC106	**+**	**+**	**+**
*E*. *coli*	MTCC1610	**-**	**-**	**+**
*V*. *cholerae*	MTCC3904	**-**	**-**	**+**
*P*. *aeruginosa*	MTCC1934	**-**	**-**	**+**
*S*. *marcescens*	MTCC97	**-**	**-**	**+**
*C*. *freundii*	MTCC1658	**-**	**-**	**+**
*A*. *faecalis*	MTCC3104	**-**	**-**	**+**
*A*. *baumannii*	MTCC1425	**-**	**-**	**+**
*P*. *vulgaris*	MTCC1771	**-**	**-**	**+**
*C*. *albicans*	MTCC1637	**-**	**-**	**-**
*Asperigillus terreus*	Lab isolate	**-**	**+**	**-**

### Characterization of bacteriocin-like peptide

**Figure 3:**
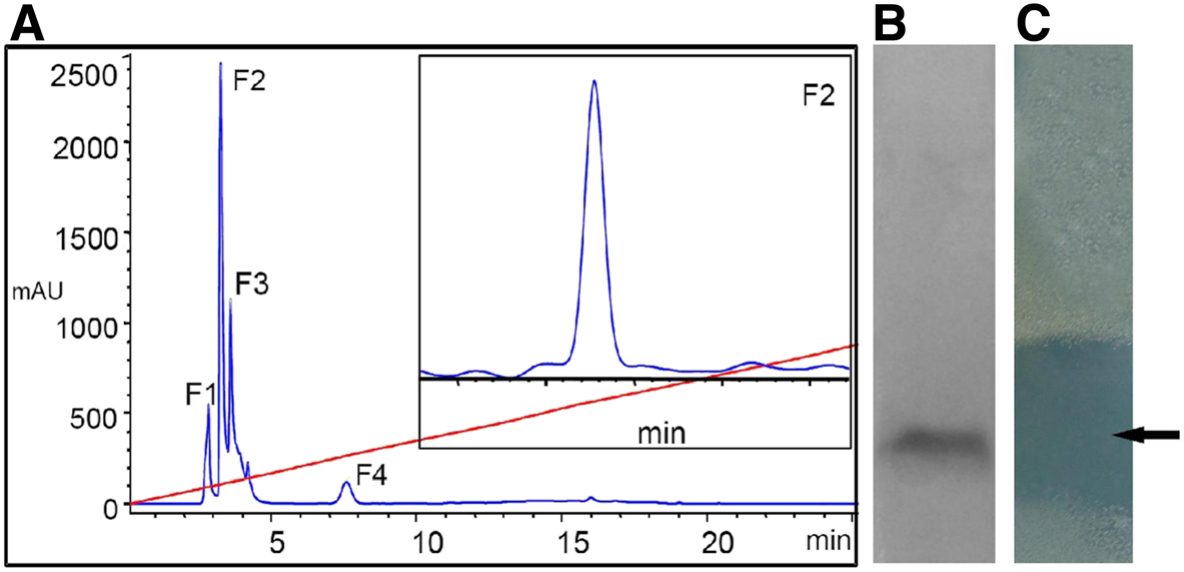
**Separation of the antimicrobial lipopeptide from acidic methanol extract by using reversed-phase HPLC.** (**A**) Chromatogram profile of acidic methanol extract showed four peaks (F1-F4) and among these fractions F2 (lipopeptide) and F4 (bacteriocin-like peptide) showed antimicrobial activity. Diagonal red line indicates the gradient of solvent B; inset picture shows the re-chromatogram profile of lipopeptide fraction (F2). (**B**) Tricine–SDS-PAGE of the purified bacteriocin-like peptide. (**C**) Direct overlay of the SDS-PAGE gel demonstrating a clear inhibition zone against test strain *S*. *aureus* (indicated with arrow).

**Figure 4:**
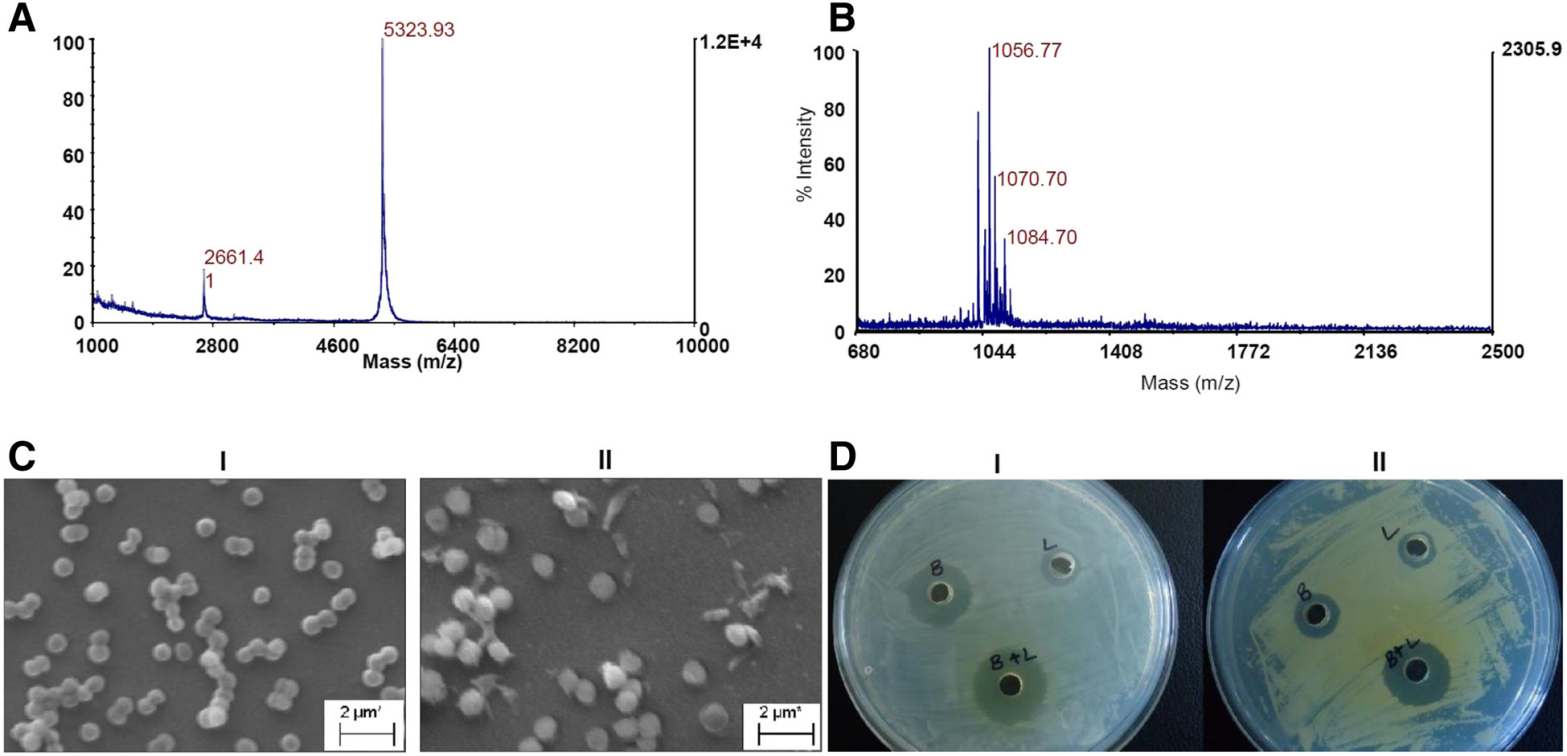
**MALDI-TOF mass spectrum of bacteriocin-like peptide (A) and lipopeptide (B) obtained by solvent extraction.** Spectrum was acquired in positive ion linear mode and reproducibility of the spectrum checked several times with different spots of same sample. (**C**) Scanning electron micrograph of *S*. *aureus* MTCC1430 cells (**I**) without treatment of bacteriocin-like peptide and (**II**) after treatment with bacteriocin-like peptide (6.0 μM) for 4 h. (**D**) Antibacterial activity of the peptides using well diffusion assay. After each peptide was serially diluted 100 μl of the lowest dilution inhibiting test strain were used for bacteriocin-like peptide (**B**), lipopeptide (**L**) and 50 μl of same dilutions were used to check the combined effect (**B+L**) on *L*. *monocytogenes* (**I**) and *M*. *luteus* (**II**). The bacteriocin-like peptide showed 14 and 12 mm inhibition zone for *L. monocytogenes* and *M*. *luteus* respectively. Lipopeptide yielded 11 mm zone for both strains. However, additive effect of both peptides showed a zone of 17 and 15 mm for *L*. *monocytogenes* and *M*. *luteus* respectively. Similar results were obtained in three individual experiments.

**Figure 5:**
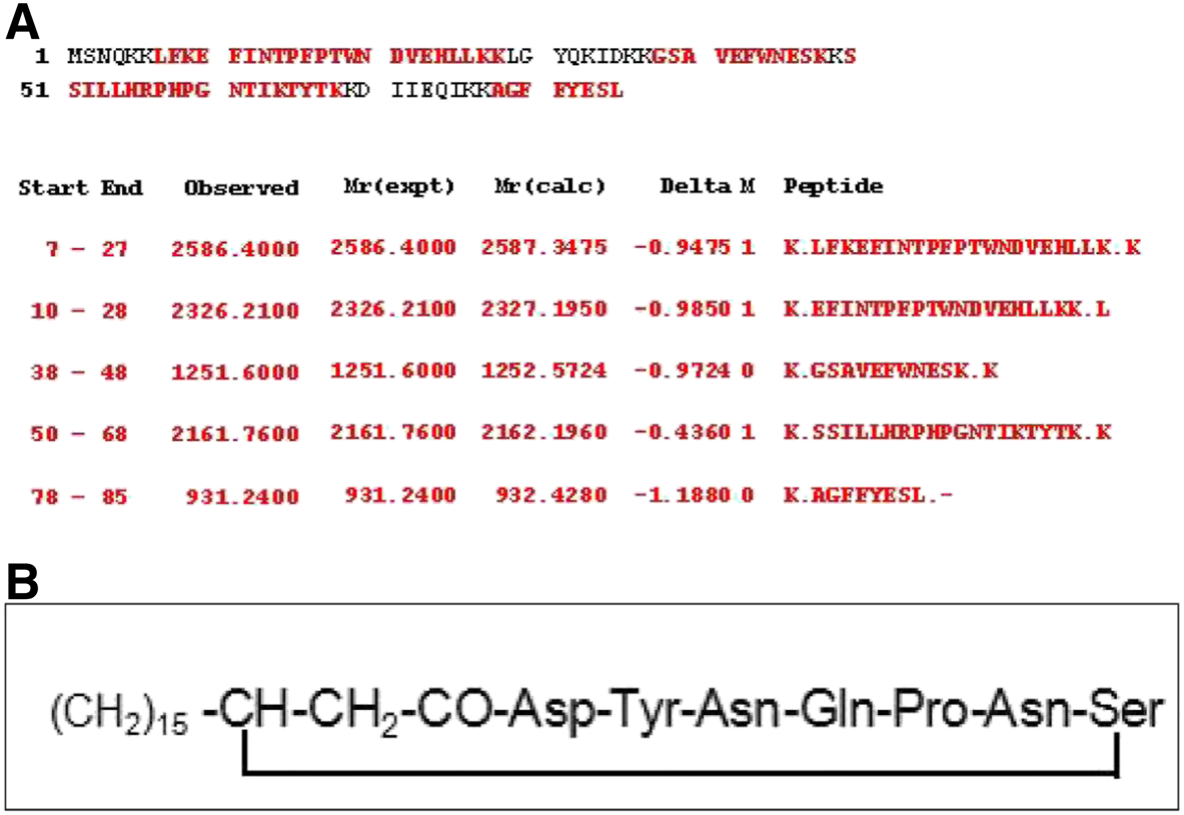
**(A) Peptide mass fingerprinting (pmf) analysis of bacteriocin-like antimicrobial peptide (5324 Da).** The pmf followed by MASCOT (Matrix Science, London, UK) search shows the matched amino acid residues (in red bold) of the peptide fragments with the putative periplasmic or secreted lipoprotein [*Methylomicrobium album* BG8] following NCBI blast search. (**B**) Amino acid sequence of the lipopeptide obtained by solvent extraction.

The peptide obtained by affinity chromatography was purified by RP-HPLC and used to determine molecular mass by MALDI-TOF analysis. Additionally, the antimicrobial peptide obtained during the lipopeptide extraction (fraction 4; Figure 3A) yielded single band in SDS-PAGE analysis (Figure 3B) and showed antimicrobial activity against *S*. *aureus* in in-gel assay (Figure 3C). Therefore, it was also included for mass determination. Both peptides showed molecular mass of 5323.9 Da (+1) and 2621.4 Da (+2) of antimicrobial peptide (Figure 4A), suggesting that it is a bacteriocin-like peptide. Further, the bacteriocin-like peptide was subjected to tryptic digestion. After tryptic digestion, the pmf analysis was performed by Matrix Science Mascot UK software, and significant (P< 0.05) result was obtained. The analysis revealed that five peptide sequences have similarity with the stretches of secreted lipoprotein of *Methylomicrobium album* BG8 (Protein View: gi|381152247) and displayed 70% coverage with a significant homology (Figure 5).

### Inhibition spectrum and sensitivity of the bacteriocin-like peptide

**Figure 6:**
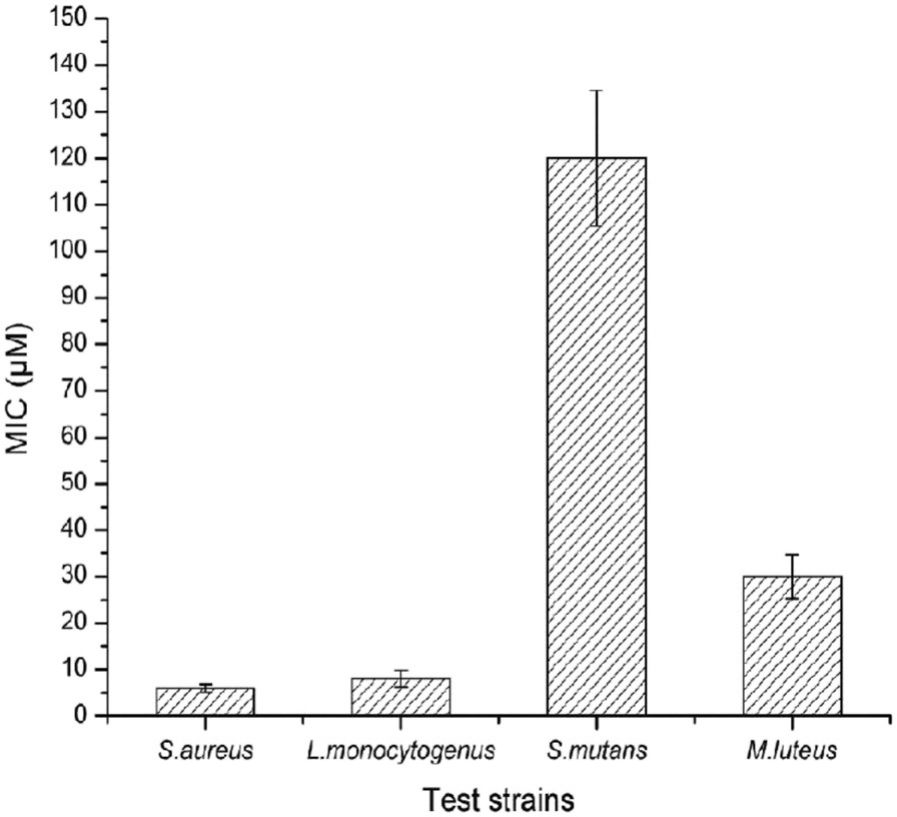
**Determination of MIC for purified bacteriocin-like antimicrobial peptide towards different Gram-positive indicator strains.** The micro-titre plate assay performed in triplicates revealed that *L*. *monocytogenes* and *S*. *aureus* are highly sensitive.

The antimicrobial peptide displayed a narrow spectrum activity and was active against Gram-positive bacteria such as *L*. *monocytogenes* (MTCC839), *S*. *aureus* (MTCC1430), *S*. *mutans* (MTCC497) and *M*. *luteus* (MTCC106). The scanning electron microscopy (SEM) of *S*. *aureus* (MTCC1430) treated with 6.0 μM concentration of bacteriocin-like peptide showed accumulation of cell debris in the medium (Figure 4C). The MIC analysis of purified peptide revealed *S*. *aureus* and *L*. *monocytogenes* are more sensitive strains and are inhibited at lower concentrations of the peptide compared to other test strains (Figure 6). No activity could be detected when tested for other Gram-negative bacteria included in the assay. The results of heat stability assay of the antimicrobial peptide shows that the antimicrobial activity is retained as high as 80°C for 15 min. Clearly, there was sharp decrease in activity with increase in temperature up to 100°C and no activity observed when the peptide was autoclaved (121°C for 15 min). The peptide did not show a change in its activity profile between pH 3.0 - 9.0, however, the activity sharply reduced to almost undetectable level above pH 9.0. No activity was observed at pH 2.0 and 10.0. Significant decrease in antimicrobial activity was observed when the peptide was incubated with pepsin, trypsin, chymotrypsin, pronase E and proteinase K for 6 h. Complete loss of antimicrobial activity was observed with overnight incubation of the peptide with above proteases. Interestingly, the antimicrobial peptide showed increase in activity when combined with lipopeptide produced by strain SK.DU.4 (Figure 4D).

### Lipopeptide purification and MS/MS sequencing

**Figure 7:**
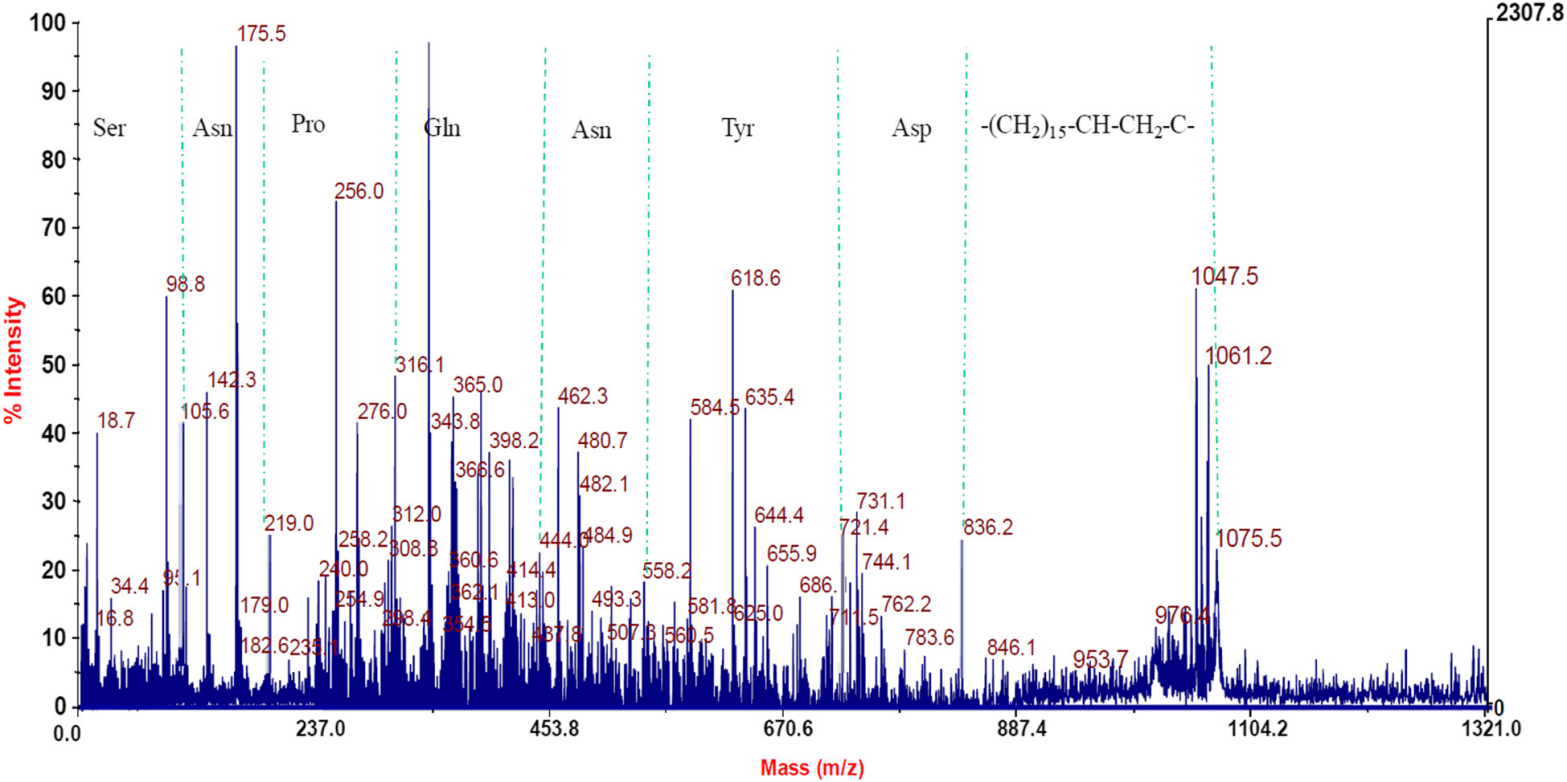
MALDI TOF PSD (MS/ MS) spectrum of lipopeptide (fraction 2 obtained in RP-HPLC of solvent extract).

In contrast to bacteriocin-like peptide, the lipopeptide from liquid culture-grown supernatant was extracted as methanolic extract and separated using RP-HPLC. Four fractions were observed during HPLC separation (Figure 3A). After lyophilization, all fractions were tested for antimicrobial activity and found that fractions 2 and 4 exhibiting antimicrobial activity. Fraction 4 was identified as bacteriocin-like peptide as described above and fraction 2 showed a low molecular mass of 1056 Da (Figure 4B). The primary structure elucidation of the fraction 2 peptide was made using a combination of mass spectrometry techniques. The peaks obtained for different fragments at m/z 836, 721, 558, 444, 316, 219, 105 and 18 in MS/MS analysis revealed the lipopeptide sequence as Asp-Tyr-Asn-Gln-Pro-Asn-Ser (Figure 7). The C-terminal amino acid in peptide was linked to hydroxyl group of a β-hydroxy-fatty acid through lactone ring, a characteristic feature observed for cyclic lipopeptides. Based on the amino acid composition, fraction 2 lipopeptide was identified as member of the iturin family biosurfactants with a mass value of m/z 1056 Da.

## Discussion

Antimicrobial peptides are produced as defensin molecules by many Gram-positive bacteria including *Bacillus* in complex environments. Habitats like rhizosphere provide competitive environment resulting in multiple strains producing antimicrobial peptides that exhibit selective spectrum of inhibition. In addition to antimicrobial peptides, many strains of bacteria are reported to produce a variety of antimicrobial biosurfactants like lipopeptide antibiotics, classified as iturins or surfactins (Chen and Hoover 2003). Screening and characterization of these novel antimicrobial peptides has attracted attention of many researchers due to their potential applications in therapeutic and food industry. Moreover, combined production of antimicrobial peptide and bioactive lipopeptide by a single strain can import additional benefits to the strain(s) for commercial applications like food preservation. Likewise, few strains of *Bacillus subtilis* known to produce various antimicrobial peptides including lipopeptides with potential applications (Toure et al. 2004; Chen et al. 2008) and in few instances co-production of a variety of lipopeptides like surfactin/iturin or surfactin/mycosubtilin have been reported from *Bacillus* sp. (Huang et al. 1993; Duitman et al. 1999). However, co-production of bacteriocin-like antimicrobial peptide and a lipopeptide has never been reported for any strain of the genus *Bacillus*. In the present study, we report a novel strain SK.DU.4, identified during screening of soil bacteria for antimicrobial substances production, which produces both bacteriocin-like peptide and lipopeptide. Although strain SK.DU.4 showed highest similarity with *B*. *subtilis* subsp. *inaquosorum* (99.8% similarity) during blast search of 16S rRNA gene sequence, it differed in phenotypic properties such as presence of ellipsoidal endospore and growth at 14% NaCl concentration with the closest phylogenetic relative *B*. *subtilis* subsp. *inaquosorum*. In addition, the lipopeptide molecular mass of strain SK.DU.4 differed with surfactin-like lipopeptide (1120.8 m/z) produced by *B*. *subtilis* subsp. *inaquosorum* (Rooney et al. 2009). Further characterization of the antimicrobial peptides produced by strain SK.DU.4 revealed that it produced a bacteriocin-like peptide and an iturin like lipopeptide. Although production of multiple peptides by a single strain imparts biotechnological potential to the producing microorganism, purification of the peptides involves additional complications. However, the purification of bacteriocin like antimicrobial peptide in this study was achieved by a combination of size exclusion, affinity and reversed phase chromatography techniques as described previously (Singh et al. 2012). In contrast, the lipopeptides which exist as homologues with marginal differences in their physicochemical properties (Wang et al. 2004) were solvent extracted from liquid culture-grown supernatant (Vater et al. 2002) and subsequently purified by reversed phase chromatography.

The pmf analysis of 5.3 kDa antimicrobial peptide revealed high similarity with the lipoprotein of *Methylomicrobium album*, a secreted hydrolytic enzyme that are known to degrade the structural components of the cell wall and thereby exhibiting antimicrobial activity (Collmer and Keen 1986). Similarly, disintegration of the cell wall might be the cause of antimicrobial activity of the bacteriocin-like peptide as cell debris observed in the surroundings of the treated cells (Figure 4C II). Nevertheless, no lipid moiety was detected in TLC experiments carried out for the detection of total lipids of the antimicrobial peptide. However, characteristics like the low-molecular-weight, narrow spectrum antimicrobial activity against Gram-positive bacteria, sensitivity to proteolytic enzymes and resistance to high temperature identified the peptide as a bacteriocin-like antimicrobial peptide belonging to class II nonmodified peptides (Klaenhammer 1993). Strains of the genus *Bacillus* produces a wide variety of bacteriocins that are encoded by a cluster of genes comprising structural, transportation, immunity, regulatory and other genes required for post-translational modifications (Le Marrec et al. 2000; Lee et al. 2009; Abriouel et al. 2011). Antimicrobial peptides produced by members of the genus *Bacillus* are classified under both bacteriocins with post-translational modifications and nonmodified antimicrobial peptides (Abriouel et al. 2011). However, peptide mass fingerprint analysis of the peptide in this study yielded different fragments (Figure 6) and the bioinformatic analysis of one of the largest peptide fragment sequence (KLFKEFINTPEPTWNDVEHLLKK) showed 34% hydrophobicity. The motif –LFKEFI- at N-terminal site of the sequence is interesting where KE, the hydrophilic residues are surrounded by hydrophobic residues (LF and FI) are might be important for cell penetration and cell membrane interaction. Furthermore, the presence of a bioactive lipopeptide peak in RP-HPLC analysis of solvent extract (Figure 3A) revealed that the strain SK.DU.4 also produced an antimicrobial surfactant. Antimicrobial lipopetides are known to produce by various strains of *B*. *subtilis* and they includes iturin, bacillomycin, mycosubtilin and fengycin with molecular weight ranging between 1028–1084 Da and exhibit strong antimicrobial activity towards many pathogenic fungi (Vanittanakom et al. 1986; Duitman et al. 1999; Yu et al. 2002; Chen et al. 2008). These amphipathic lipopeptides are non-ribosomal peptides biosynthesized by large multi-enzymes called non-ribosomal peptide synthetases (NRPSs) with modularly arranged catalytic domains (Peypoux et al. 1999; Finking and Marahiel 2004). Therefore, to probe the composition and activity of the isolated lipopeptide, its primary structure was determined using a combination of chemical reactions and a variety of mass spectrometry techniques. Based on the presence of β-hydroxy fatty acid moiety, hepta peptide and its amino acid composition, the lipopeptide was identified as a member of the iturin family. However, it exhibited stronger antibacterial activity than antifungal activity when compared with other members of the iturin family. Additionally, strain SK.DU.4 contained aspartic acid (D) compared to asparagine (N) in iturins and mycosubtilin in their N-terminal consensus sequence Asx-Tyr-Asn. Though, the sequence of Asp-Tyr-Asn was observed at N-terminal for bacillomycin (Peypoux et al. 1986), it differed with lipopeptide of strain SK.DU.4 in the composition of other amino acids. The lipopeptide was not tested for any surfactant activity as there are no hydrophobic amino acids observed in the sequence. Lipopeptides easily bind to the bacterial surface bilayer and alter the local lipid organizational linking on negatively charged fatty acids resulting in restructuring of the lipid bilayer and prevent cellular processes (Horn et al. 2012). The fatty acid moiety is also found to play an important role in antimicrobial activity of lipopeptides (Boeck et al. 1989) and members of the iturin family lipopetides including iturin A, bacillomycin D, bacillomycin F, bacillomycin L and bacillomycin Lc, contained a β-hydroxy fatty acid with 14 carbon chain length and inhibited different species of fungi but exhibited limited activity against bacteria (Vanittanakom et al. 1986; Zeriouh et al. 2011). In comparison, the lipopeptide in this study contained a β-hydroxy fatty acid of 15 carbon chain length and showed stronger antibacterial activity. Although the lipopeptide inhibited growth of *B*. *subtilis* (MTCC121), it did not inhibit the growth of other *B*. *subtilis* strains including producing strain SK.DU.4. Lipopeptide also inhibited the growth of strains like *S*. *mutans* in combination with bacteriocin-like peptide. Thus, the additive effect of both peptides exhibited increase in antimicrobial activity against Gram-positive bacteria.

In conclusion, strain designated as SK.DU.4, identified as *Bacillus subitlis* produced two novel antimicrobial peptides that are active against a variety of Gram-positive bacteria. The co-production of these antimicrobial peptides by strain SK.DU.4 increased its applications as a biocontrol agent or in food preservation due to its high antimicrobial activity against *S*. *aureus* and *L*. *monocytogenes*, which are frequently connected to food spoilage.

## Competing interest

The author(s) declare that they have no competing interests.
